# Waterless structures in the Protein Data Bank

**DOI:** 10.1107/S2052252524009928

**Published:** 2024-10-28

**Authors:** Alexander Wlodawer, Zbigniew Dauter, Pawel Rubach, Wladek Minor, Joanna I. Loch, Dariusz Brzezinski, Miroslaw Gilski, Mariusz Jaskolski

**Affiliations:** a Center for Structural Biology National Cancer Institute Frederick MD21702 USA; b Department of Molecular Physiology and Biological Physics University of Virginia Charlottesville Virginia USA; c Warsaw School of Economics Warsaw Poland; d Department of Crystal Chemistry and Crystal Physics, Faculty of Chemistry Jagiellonian University Krakow Poland; e Institute of Computing Science Poznań University of Technology Poznań Poland; f Institute of Bioorganic Chemistry Polish Academy of Sciences Poznań Poland; g Department of Crystallography, Faculty of Chemistry Adam Mickiewicz University in Poznań Poznań Poland; University of Auckland, New Zealand

**Keywords:** protein hydration, structure-factor statistics, structure-quality statistics, modeling errors, electron density, Protein Data Bank, *PDB-REDO*, protein structure, X-ray crystallography

## Abstract

The absence of solvent molecules in high-resolution protein crystal structure models deposited in the Protein Data Bank (PDB) contradicts the fact that, for proteins crystallized from aqueous media, water molecules are always expected to bind to the protein surface, as well as to some sites in the protein interior. An analysis of the contents of the PDB indicated that the expected ratio of the number of water molecules to the number of amino-acid residues exceeds 1.5 in atomic resolution structures, decreasing to 0.25 at around 2.5 Å resolution. Nevertheless, almost 800 protein crystal structures determined at a resolution of 2.5 Å or higher are found in the current release of the PDB without any water molecules, whereas some other depositions have unusually low or high occupancies of modeled solvent. Detailed analysis of these depositions revealed that the lack of solvent molecules might be an indication of problems with either the diffraction data, the refinement protocol, the deposition process or a combination of these factors. It is postulated that problems with solvent structure should be flagged by the PDB and addressed by the depositors.

## Abstract 2

A global analysis of protein crystal structures in the Protein Data Bank reveals many medium-to-high-resolution entries that lack any solvent molecules. Also, there are many cases with impossible occupancies of water molecules and/or uninterpreted very high difference electron-density peaks that indicate the presence of unmodeled water molecules.

## 1. Introduction

As is often the case, the origins of this project are quite incidental. When analyzing the highest-resolution crystal structure of crambin from the point of view of the complete solvent structure (Chen *et al.*, 2024 ▸), we were perplexed by the observation that several crystal structures of this protein, a champion of ultimate resolution among macromolecules, are present in the Protein Data Bank (PDB; Burley *et al.*, 2018 ▸) without solvent molecules. This is true even when the title and primary reference explicitly refer to patterns formed by water molecules (Teeter, 1984 ▸). Intrigued by this observation, we conducted a global search of the PDB holdings, looking for medium-to-high-resolution protein crystal structures in which water molecules were not characterized. We found hundreds of such cases. Among the incriminated depositions, there were crystal structures with abnormally high *R* factors (>0.5) or erroneous relations between *R* and *R*_free_ (*R* > *R*_free_). Moreover, our analysis of structures re-refined by *PDB-REDO* (Joosten *et al.*, 2011 ▸) shows similar problems despite the latest software being used to produce improved models of macromolecular structures. At the same time, some of us were also investigating the quality of l-asparaginase structures in the PDB, especially from the point of view of agreement between the model and the electron density (Wlodawer *et al.*, 2024 ▸). The tool that we developed for the automatic identification of difference electron-density peaks in the structures of l-asparaginases was then applied against the contents of the whole PDB, retrieving many additional entries for which the *mF*_o_ − *DF*_c_ (hereafter abbreviated as *F*_o_ − *F*_c_) maps had very large and uninterpreted peaks (positive or negative). These procedures defined the framework of the present study.

With very rare exceptions, proteins are crystallized from aqueous solutions. Well ordered water molecules are expected to bind to the protein surface and often in internal cavities. Thus, it would be expected that each globular protein structure determined at medium-to-high resolution should contain at least some ordered solvent molecules. Integral membrane proteins might not behave in the same way, but only three such structures that do not contain water molecules (PDB entries 1vf7, 4rng and 7udy) passed our search criteria, so they do not affect our analysis in a significant way. We understood that, for technical reasons, solvent molecules may not be detectable in low-resolution structures (here defined as lower than 2.5 Å; see below). In this manuscript, we follow in the footsteps of previous critical analyses of the quality of macromolecular structures, as exemplified by the iconic publications by the Uppsala group (Kleywegt & Jones, 1995 ▸, 1996 ▸; Kleywegt *et al.*, 1996 ▸).

## 2. Materials and methods

### 2.1. Data acquisition and processing

The data used in this analysis were extracted from the PDB on 14 May 2024. The presence of water molecules in each deposition was established based on the parameter ‘Number of Water Molecules per Deposited Model’. Of the total of 219 792 entries that were present in the database, 47 831 did not contain any water molecules. We selected a significantly reduced set of waterless structures for further analysis by applying other filters. Our working set was limited to only protein crystal structures determined using X-ray or neutron diffraction, with polypeptide chains consisting of at least 30 amino-acid residues. We excluded all structures with resolution lower than 2.5 Å and all entries labeled ‘Group deposition’. The final working data set consisted of 787 structures, 554 of which were accompanied by structure factors (Supplementary File S1).

To analyze the quality of structures without water molecules and to compare them against the rest of the PDB, we gathered data from PDB validation reports and implemented dedicated scripts to calculate statistics from electron-density maps (Supplementary File S2). Data taken directly from the PDB depositions and also from the validation reports were used to extract parameters such as *R*_merge_ (treated as equivalent to *R*_scale_), *R*, *R*_free_, low- and high-resolution limits, number of free reflections, clashscore, Ramachandran outliers, rotamer outliers, real-space *R*-value *Z*-score (RSRZ) outliers, root-mean-square *Z*-score (RMSZ) for bond lengths, RMSZ for angles, bulk-solvent *B* and *k*, Wilson *B* factor, percentage solvent content, number of non-H atoms, number of amino-acid residues, number of water molecules and molecular weight. The highest peaks in the *F*_o_ − *F*_c_ difference map (positive and negative) were identified for each of the structures with experimental data available. We used *Coot* (Emsley *et al.*, 2010 ▸) to detect all peaks with a height above 5.0σ, calculated based on the data extracted from the MTZ files downloaded from the PDB. These searches provided the initial estimate of the number of unassigned water molecules, and selected maps were also analyzed visually to validate the general procedures. Subsequently, the corresponding 2*mF*_o_ − *DF*_c_ (hereafter abbreviated as 2*F*_o_ − *F*_c_) electron-density maps were searched using the *Coot* ‘Find waters’ procedure. Newly identified putative water molecules were added at unoccupied sites at the positions of 2*F*_o_ − *F*_c_ peaks above the 1.4σ level. Such altered structural models were then refined using *REFMAC*5 (Murshudov *et al.*, 2011 ▸), which was run in an automated manner.

### 2.2. Statistical and data-mining methods

We employed several statistical and data-mining techniques to analyze the properties of waterless structures in the PDB. To verify whether waterless structures can be categorized into groups, we used agglomerative hierarchical clustering (Tan *et al.*, 2005 ▸) with Ward linkage (Ward, 1963 ▸). Since many quality metrics were missing from the PDB depositions and/or validation reports, missing values were addressed through median imputation for numerical stability. The data were subsequently scaled using a 0–1 min–max normalization to ensure equal importance of all structure-quality metrics. This preprocessing step was crucial to eliminate biases arising from different measurement scales.

Outliers among waterless structures were detected using the local outlier factor (LOF; Breunig *et al.*, 2000 ▸) method with *k* = 5 neighbors, using the same median-imputed 0–1 scaled data as for clustering. The LOF is particularly useful for identifying anomalies in the data based on the local density deviation of a given data point with respect to its neighbors. This method helped us to isolate depositions among waterless structures that significantly deviate from the norm.

To visually compare waterless structures against other depositions in the PDB, we performed principal component analysis (PCA) dimensionality reduction (Jolliffe, 2002 ▸). Since PCA is sensitive to missing values and data imputation, for this part of the analysis we decided to use only depositions with all quality metrics available (52 213 depositions). Moreover, before PCA, the data were standardized to ensure that each variable contributed equally to the analysis.

## 3. Results

A search of the PDB identified 787 depositions refined with diffraction data extending to 2.5 Å resolution or higher that did not contain any water molecules (Supplementary File S1). These structures are evaluated below. However, since the original impetus for this study was provided by the observation that a number of structural depositions found in the PDB for the small protein crambin (46 residues) do not contain any associated solvent, these structures will be analyzed and discussed first.

### 3.1. The strange case of crambin structures

Crambin and its close relatives readily yield highly diffracting crystals, resulting in several depositions in the PDB at ultrahigh resolution. We were surprised to find that a number of crambin structures had been deposited without the inclusion of water molecules (PDB entries 1ejg, 1crn, 1cnr, 1cbn, 1ab1, 1jxt, 1jxu, 1jxw, 1jxx and 1jxy). The experimental details for all of them have been described in accompanying publications (Teeter, 1984 ▸; Jelsch *et al.*, 2000 ▸; Yamano & Teeter, 1994 ▸; Teeter *et al.*, 1993 ▸, 2001 ▸; Yamano *et al.*, 1997 ▸). We understand that some of these manuscripts were focused on a particular problem and the status of water molecules might be of secondary importance. However, in some cases the solvent structure was the focal point of the study (and of the publication; Teeter, 1984 ▸). These structures have very high to ultrahigh resolution; thus, the contribution of solvent molecules is very important for many follow-up studies. We inspected these depositions to confirm that some statistical irregularities are not the result of a flawed deposition process.

We are puzzled why the depositions listed above do not contain water molecules, which are an integral component of these protein structures. Even if a specific effect, for example, crystal vitrification temperature, is investigated, one needs to remember that the crystal is composed of protein, additives (buffer, cofactor *etc.*) and solvent. In the X-ray diffraction experiment, the entire atomic model must be considered as a whole, and its parts cannot be separated *ad lib*. Otherwise the reported results do not correctly describe the experimental conditions and refinement procedures.

In PDB entries 1jxt, 1jxu, 1jxw, 1jxx and 1jxy high peaks are present in the *F*_o_ − *F*_c_ and 2*F*_o_ − *F*_c_ electron-density maps. These peaks are well defined and unambiguously correspond to missing water molecules. In contrast, in PDB entry 1ejg water molecules have been removed from the model used for the calculation of the phases present in the MTZ file deposited in the PDB.

It is puzzling that the title of entry 1crn in the PDB is ‘*Water structure of a hydrophobic protein at atomic resolution. Pentagon rings of water molecules in crystals of crambin*’ and the same title was used for the relevant manuscript (Teeter, 1984 ▸), yet no water molecules are present in this deposition. Structure factors for this deposition are not available in the PDB; thus, a visual inspection of the electron density could not be performed. For the same reason, this structure is not present on the *PDB-REDO* website. Furthermore, there is a huge discrepancy between the resolutions reported in the PDB deposition and in the reference publication (1.5 versus 0.945 Å).

A similar situation was found with two other structures, PDB entries 1cnr (Yamano & Teeter, 1994 ▸) and 1cbn, for which experimental data are again unavailable. For PDB entry 1ab1, representing the Ser22/Ile25 variant of crambin, the correlation of the side chains of these two residues and the water structure is described (Yamano *et al.*, 1997 ▸), although no water molecules are present in the deposition.

In August 2023, we alerted the PDB about the problem with missing water molecules in crambin depositions and, after prolonged deliberations among the wwPDB partners, received an answer in November 2023 that ‘OneDep will check for entries that do not contain waters and will provide a warning message to alert depositors that a statement of this will appear in the validation report’ and that ‘Biocurators will contact the authors of existing X-ray entries with a resolution higher than 2.0 Å that do not have waters to suggest coordinate replacement’.

### 3.2. The expected number of water molecules in deposited protein structures

**Figure 1:**
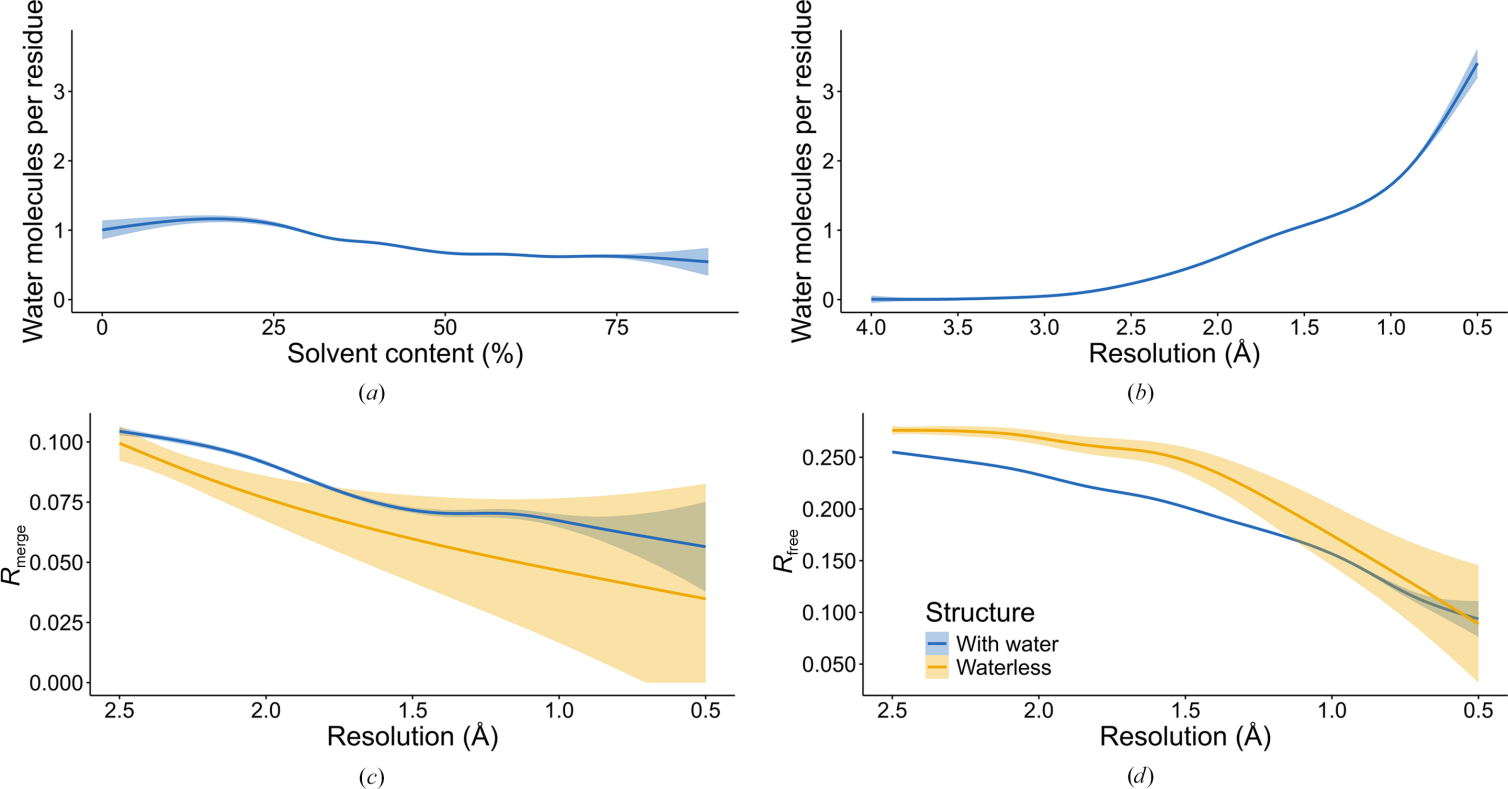
Comparison of PDB protein structures without (yellow) and with (blue) water molecules determined at a resolution of 2.5 Å or higher. The solid lines represent the piecewise cubic regression (splines) fitted to the data; the shaded area represents the 95% confidence interval of the regression line. (*a*) The average ratio of the number of water molecules to the number of amino-acid residues for varying percentage solvent contents. (*b*) The average ratio of the number of water molecules to the number of amino-acid residues as a function of the resolution of the diffraction data. (*c*) *R*_merge_ as a function of the resolution of the diffraction data. (*d*) *R*_free_ as a function of the resolution of the diffraction data.

The number of water molecules that can be visible in protein crystals depends on different factors. Although the volume fraction of solvent (Matthews, 1968 ▸) might be correlated with the number of water molecules present in a PDB deposition, we did not find this correlation to be significant [Fig. 1 ▸(*a*)], since it is unlikely that waters extending beyond the second shell of hydration could be reliably modeled. However, we found an expected clear correlation between the reported resolution of the structure and the number of observed water molecules, reported as the ratio between the number of water molecules and the number of protein residues [Fig. 1 ▸(*b*)]. Whereas the mean ratio is 1.78 for structures at resolutions higher than 1.0 Å, it falls to just below 0.61 at 2.0 Å and to 0.25 at 2.5 Å. The number of water molecules visualized at resolutions lower than 2.5 Å becomes negligible, an observation validating our choice of 2.5 Å as the low-resolution limit for structures to be analyzed in this study. A more detailed, interactive version of Fig. 1 ▸(*b*) that allows the identification of outliers can be found at https://bioreproducibility.org/figures/water_paper/fig1B.

The quality of diffraction data [as measured by the value of *R*_merge_ (or *R*_sym_; these parameters are used interchangeably in various depositions)] is correlated with the resolution, but by this criterion the waterless structures do not seem to be any worse than the averages for all structures in the PDB [Fig. 1 ▸(*c*)]. Other relevant parameters are related to the refinement statistics of the models, in particular *R*_free_. Fig. 1 ▸(*d*) illustrates the relationship between *R*_free_ [provided by the depositor or recalculated by the Digital Curation Centre (DCC) if the former is not available] and the resolution of the diffraction data, showing the expected increase in *R*_free_ as the resolution becomes lower. It is also very clear that the values of *R*_free_ are generally higher for the waterless structures compared with all other structures in a given resolution range.

**Figure 2:**
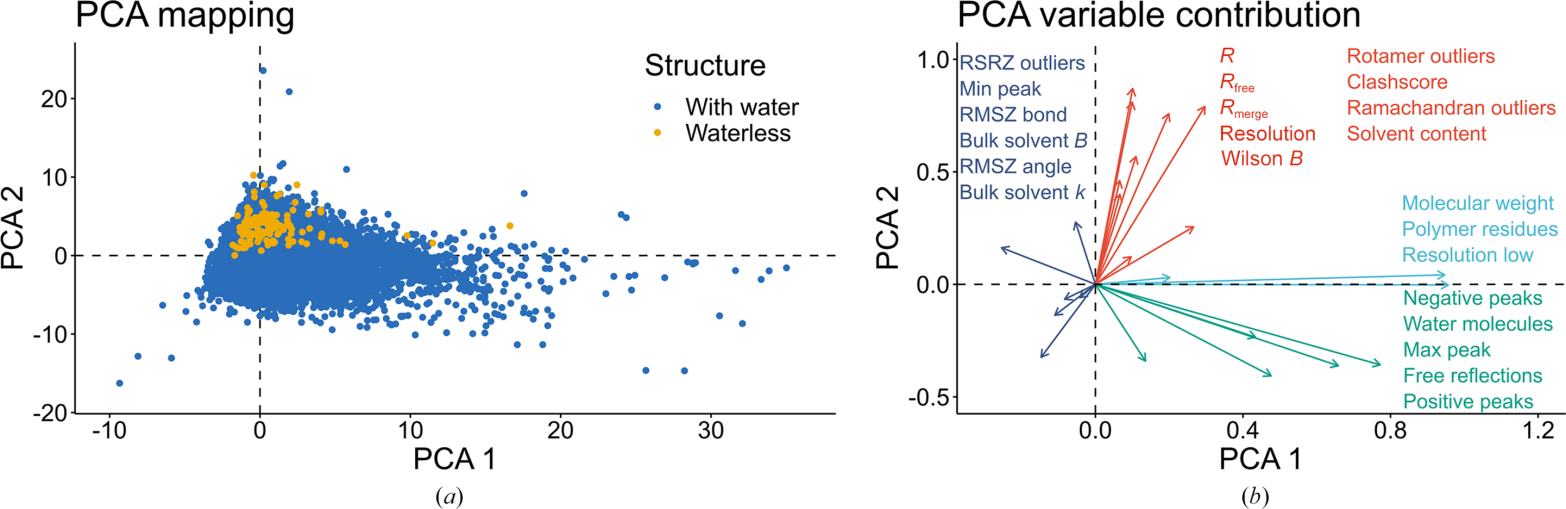
Comparison of waterless structures against structures with water present using principal component analysis (PCA). (*a*) PCA mapping of waterless structures (yellow points) and crystal structures with a resolution of 2.5 Å or higher (blue points). (*b*) PCA variable contribution plot, showing the directions of high values of the analyzed quality metrics.

To carry out a more general comparison of waterless structures against other depositions in the PDB, we performed principal component analysis (PCA) using data from the PDB entries, validation reports and our custom scripts that analyzed electron-density peaks in depositions. As can be noticed in Fig. 2 ▸, waterless structures usually contain fewer residues than other PDB depositions and have relatively poorer quality metric values (*R*_free_, clashscore, RMSZ outliers, rotamer outliers, *etc.*).

We have also verified whether waterless structures form clusters among themselves. After inspecting the results of agglomerative clustering (Supplementary Fig. S1), we did not notice any obvious clusters. However, it was clear that there are some very atypical structures among waterless depositions. Therefore, we performed outlier detection using the local outlier factor algorithm. The waterless structures with the highest outlier factors (Supplementary File S3) included PDB entries 1dqg (with an RMSZ on bond lengths of 53.96), 1ejg (a 0.5 Å resolution structure with a 33σ maximum electron-density peak in the *F*_o_ − *F*_c_ map), 1h6j (bulk-solvent *B* factor 467 Å^2^), 1hpb (with 100 rotamer outliers), 1fx1, 7xin (a 2.0 Å resolution structure with *R*_free_ around 0.50) and 2vqe (with 27 negative difference electron-density peaks <5σ and 76 positive peaks >5σ). In the following section, we discuss some of these and other interesting structures in more detail.

### 3.3. Analysis of waterless structures

#### 3.3.1. Structures with waters that were presumably lost during deposition

**Figure 3:**
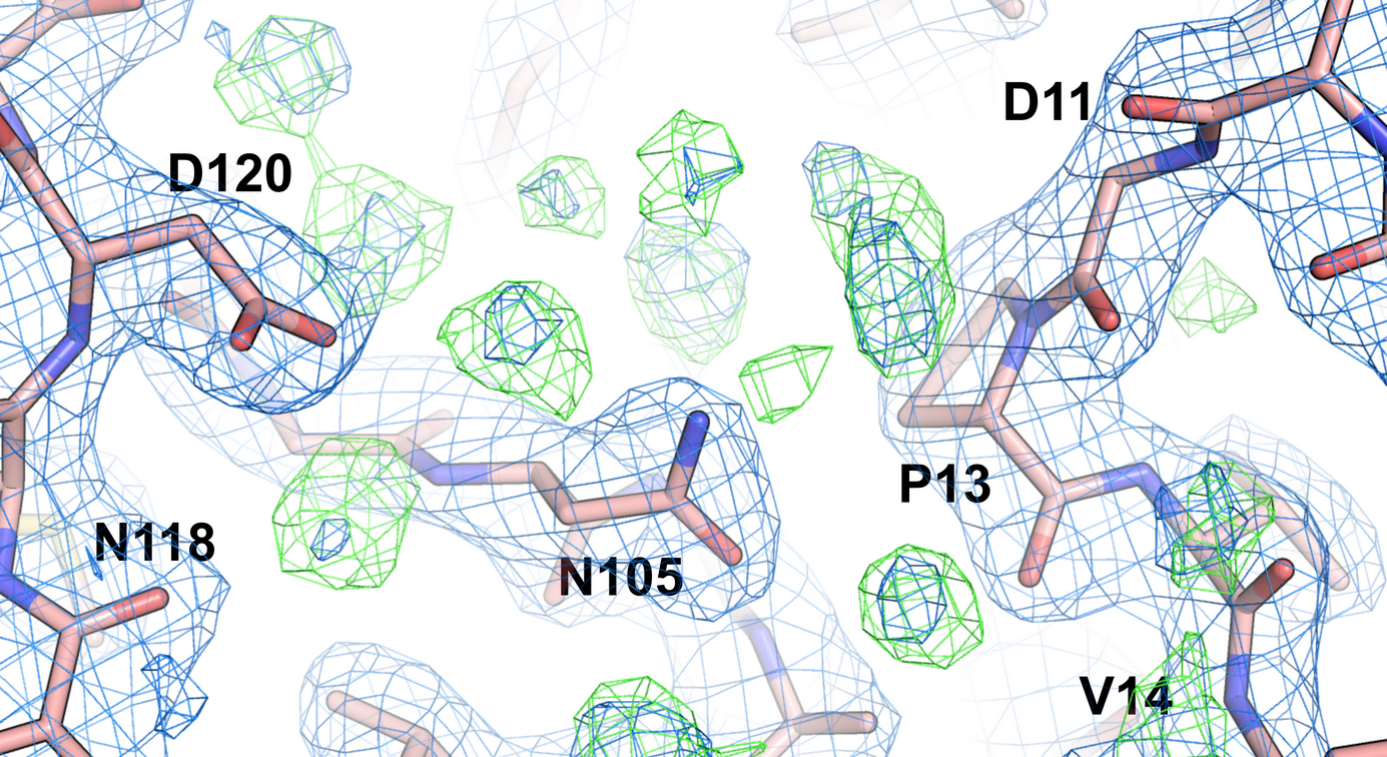
Example of unmodeled water molecules in chain *B* of the structure of a superoxide dismutase–nanobody complex (PDB entry 7nxx). A search of the *F*_o_ − *F*_c_ map using the ‘Search for solvent’ function in *Coot* identified the presence of 61 potential water molecules in this deposition. The 2*F*_o_ − *F*_c_ map (blue) is shown at the 1.5σ level, while the *F*_o_ − *F*_c_ map is shown at 3.0σ (green).

The first indication that a model that included solvent had been used in structure refinement, but that solvent was lost before or during the deposition of coordinates, may be found in the PDB validation report. Significantly lower values of *R*/*R*_free_ reported by the depositors compared with those calculated by the Digital Curation Centre (DCC; found in the validation report) strongly indicate that the structure was refined with solvent but that the values of *F*_c_ calculated by the DCC were obtained without taking solvent contribution into account, since it was missing in the deposition. In such cases, the *F*_o_ − *F*_c_ electron-density maps calculated with structure factors from MTZ files provided by the PDB yield a large number of positive peaks, indicating the inadequacy of solvent modeling. A recent example of such a case is provided by the superoxide dismutase–nanobody complex with PDB code 7nxx. This structure was determined at a resolution of 2.19 Å with *R*/*R*_free_ values of 0.202/0.245 reported by the depositors, whereas the corresponding values calculated by the DCC are 0.234/0.266. A search of the *F*_o_ − *F*_c_ map using the ‘Search for solvent’ function in *Coot* (Emsley *et al.*, 2010 ▸) identified the presence of 61 potential water molecules (Fig. 3 ▸). In this search, we used a 3.5σ electron-density cutoff in the *F*_o_ − *F*_c_ electron-density map and the presence of potential hydrogen-bond donors or acceptors within the range of 2.4–3.2 Å. Another similar case is illustrated by the structure of the PAS domain from the hEAG potassium channel (PDB entry 5j7e), with *R*/*R*_free_ values of 0.214/0.231 (depositors) and 0.241/0.250 (DCC) and with 126 water molecules found in the difference Fourier map. A more convoluted example is provided by the 1.85 Å resolution structure of cellobiose 2-epimerase (PDB entry 5zhb), for which the *R*/*R*_free_ values reported by the depositors in the validation report are 0.176/0.210. In contrast, the values from the DCC are 0.209/0.237, which roughly agree with the values of 0.193/0.236 reported by *PDB-REDO*. However, the *R*/*R*_free_ values present in the header of the PDB deposition are 0.196/0.239. In this case a search with *Coot* for water molecules resulted in 264 hits. Another similar example is provided by the crystal structure of the C19A/C43A mutant of leech carboxypeptidase inhibitor in complex with bovine carboxy­peptidase A (2.16 Å resolution; PDB entry 2abz), deposited in 2005. This deposition exhibits a large discrepancy between the *R*/*R*_free_ factors claimed by the authors and obtained by the DCC (0.189/0.234 versus 0.241/257, respectively), with as many as 211 water molecules identified by us in the *F*_o_ − *F*_c_ electron-density map. There are several other similar cases present in the PDB.

However, not all structures in which the values of the *R* factors reported by the depositors are much lower than those from the DCC could be easily explained by a lack of solvent in the deposited model coordinates. For example, the *R*/*R*_free_ values for the structure of Myxoma virus M062 protein variant Lau (2.45 Å resolution; PDB entry 7u0v) are reported as 0.211/0.266 by the depositors and 0.263/0.308 by the DCC. Nevertheless, only seven potential water sites could be seen in the *F*_o_ − *F*_c_ map. The reason for this very large *R*-factor discrepancy is clearly not related to a lack of solvent in the model.

**Figure 4:**
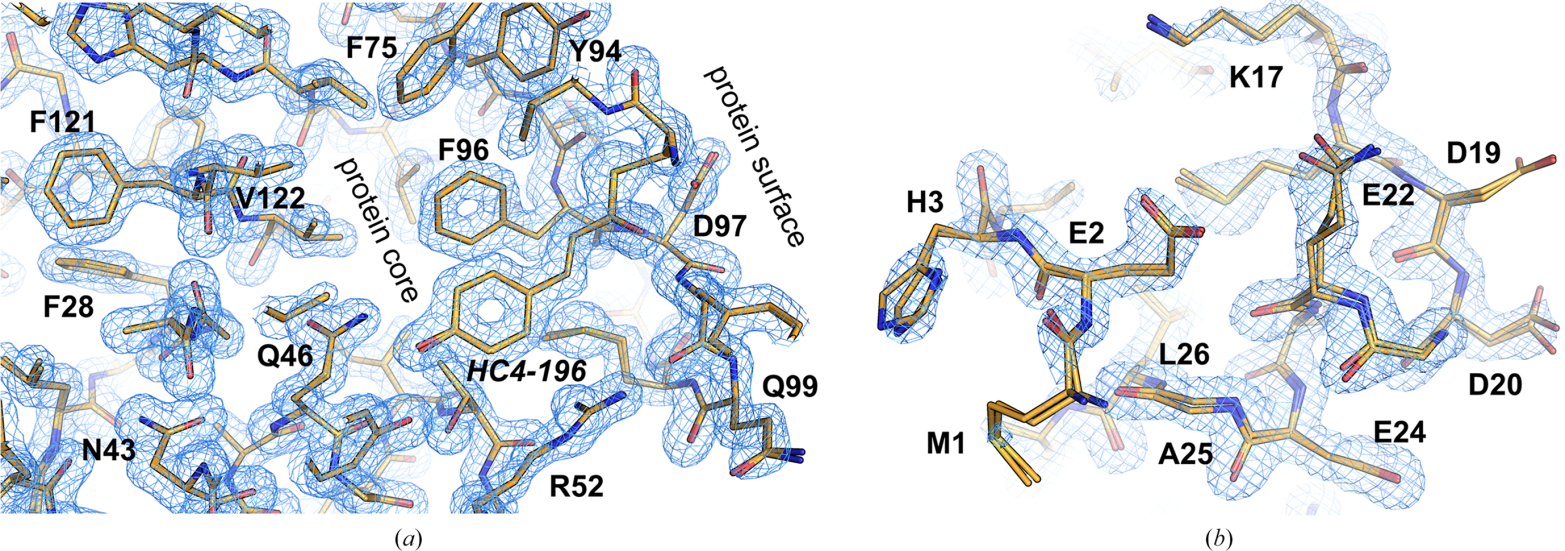
Structure of photoactive yellow protein (PDB entry 1t1c). The 2*F*_o_ − *F*_c_ map (blue mesh) is shown at the 1.5σ level. (*a*) Despite the resolution of 1.6 Å, there is no solvent in the protein core and at the protein surface even in the best areas of the map. (*b*) The less well ordered part of the map shows more clearly that the structure was modeled as two superposed molecules with occupancies of 0.71 and 0.29.

A very puzzling case is presented by the structure of photoactive yellow protein determined using Laue diffraction (PDB entry 1t1c; Rajagopal *et al.*, 2005 ▸). While the data completeness is reported to be 100%, such a value is theoretically impossible for Laue diffraction data collection (Moffat *et al.*, 1984 ▸). The crystal structure was modeled as two superposed protein molecules with occupancies of 0.71 and 0.29, and was refined with isotropic atomic displacement parameters (ADPs) at a resolution of 1.6 Å (Fig. 4 ▸). Despite the remarkably low values of *R*/*R*_free_ (0.129/0.139), there is no solvent either in the model or in the *F*_o_ − *F*_c_ electron-density map. All other structures from this series (PDB entries 1t18, 1t19, 1t1a and 1t1b), refined at the same resolution of 1.6 Å, also do not contain solvent, but their *R* factors are significantly higher (in the range of 0.22–0.31). How a structure could fit the electron density so well despite the absence of any bound solvent remains a mystery.

#### 3.3.2. Structures in which water molecules were not used during refinement

Many waterless structures, for which no attempt was made to model the solvent, have *R*/*R*_free_ values reported by the depositors that are reasonably consistent with those calculated by the DCC. Most such structures were determined at a resolution lower than ∼2.2 Å, but some higher resolution structures also fall into this category. The structure of GDSL esterase (PDB entry 8hwp), for example, determined at 1.73 Å resolution, has *R*/*R*_free_ values of 0.241/0.275 reported by the depositors and 0.252/0.285 by the DCC. A difference of ∼0.01 may be due to the use of different programs for structure-factor calculation, but the values themselves are high. As 149 unaccounted water molecules were seen by us in the *F*_o_ − *F*_c_ electron-density map, this seems to be a case of depositing a partially refined model when a better refined model could easily be made available. Interestingly, the isomorphous structure PDB entry 8hwo (1.96 Å resolution) is an extreme example of the difference between *R*/*R*_free_ values reported by the depositors (0.244/0.299) and by the DCC (0.439/0.430). In the latter case, only four water molecules could be found in the difference map, clearly indicating severe problems with the experimental data or the refinement procedures. It should also be noted that in this case *R*_free_ is lower than *R* (DCC), which is a highly awkward relationship.

The 1.9 Å resolution structure of type III antifreeze protein (PDB entry 2msi; DeLuca *et al.*, 1998 ▸) was most likely refined without modeling water molecules, although its PDB validation report is ambiguous due to a lack of marking the reflections used for cross-validation in the MTZ file. However, 22 water molecules associated with this small protein (65 residues) could be clearly seen in the *F*_o_ − *F*_c_ map and the structure could be easily re-refined. It should be noted that the coordinates resulting from re-refinement with *PDB-REDO* (Joosten *et al.*, 2011 ▸) still did not include any water molecules. As an aside, it should be mentioned that this structure was used for testing a very elaborate refinement procedure with *Amber*, and the resulting model was deposited in the PDB as entry 7q3v as an alternative to 2msi (Mikhailovskii *et al.*, 2022 ▸). The latter procedure utilized expansion of the diffraction data to space group *P*1, but practically the same 39 water molecules were placed in the resulting model as in our subsequent re-refinement (PDB entry 9cbe; Dauter & Wlodawer, 2024 ▸) that utilized a standard refinement protocol implemented in *REFMAC*5 (Murshudov *et al.*, 2011 ▸). The results of our refinement indicate that the number of water molecules from the first *F*_o_ − *F*_c_ map is underestimated, and that more water molecules are found as the structure is refined further.

In several cases of multiple, closely related structures described in a single publication, some have modeled solvent and others do not. An example of such a case is provided by the seven structures of human PPAR-α/δ (Kamata *et al.*, 2023 ▸). Whereas PDB entries 8hul (2.46 Å resolution) and 8hup (2.36 Å resolution) contain no associated water molecules (although we could identify 50 and 25, respectively, in the difference Fourier maps), water molecules are present in the higher resolution PDB entries 8huq and 8hqn (but not in PDB entries 8huk and 8huo that are at a resolution lower than 2.5 Å). Only four water molecules are present in PDB entry 8hum (2.29 Å). This example emphasizes that when multiple related structures are refined and deposited, the authors sometimes do not treat them with the same care.

### 3.4. Structures in which the presence of water molecules is not supported by the data

**Figure 5:**
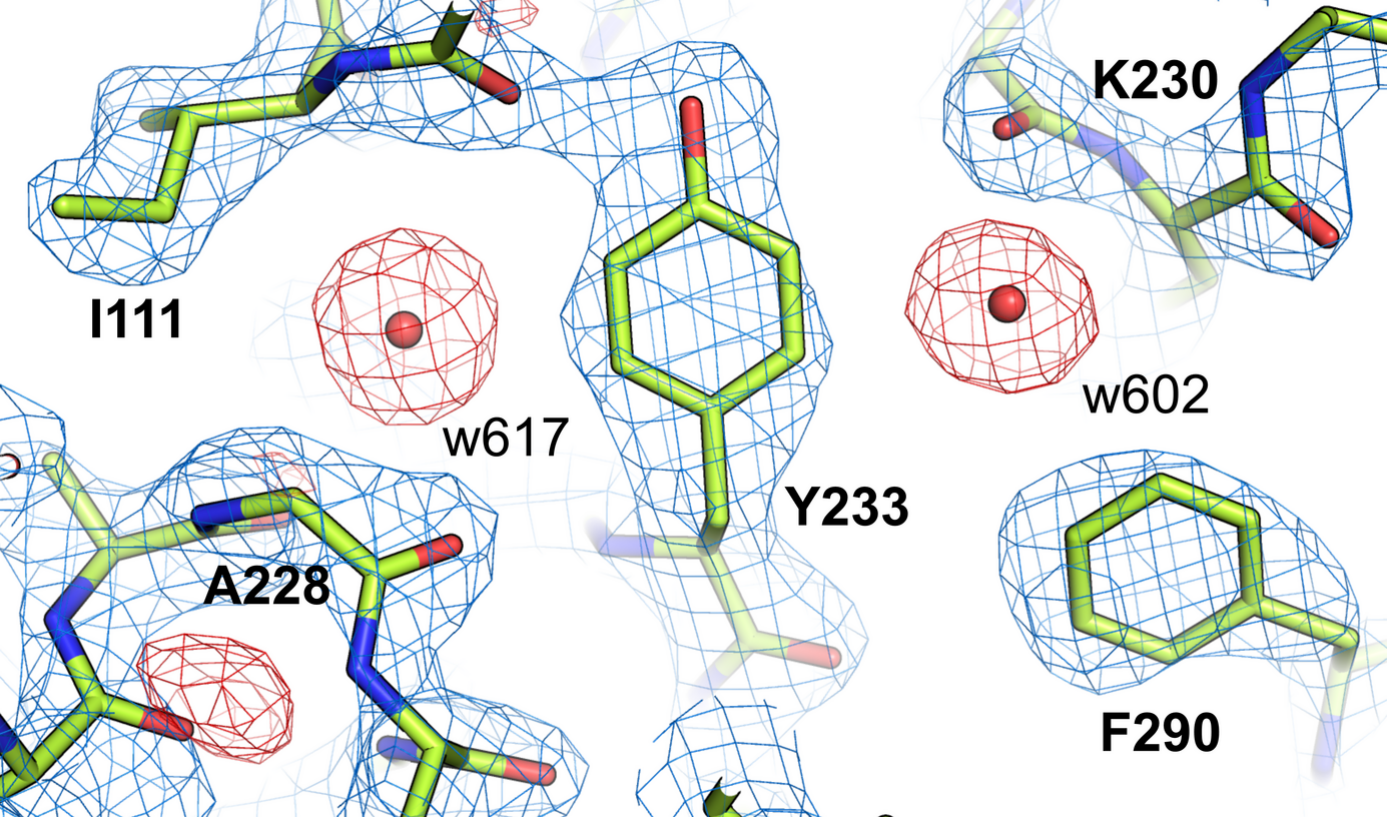
Example of a protein structure with an excess of water molecules: the botulism neurotoxin light chain (PDB entry 7kyf) contains 19 water molecules modeled at full occupancy, but all of these water molecules are located in negative regions of the *F*_o_ − *F*_c_ map, suggesting that their placement was not based on the available diffraction data. The 2*F*_o_ − *F*_c_ map (blue) is contoured at the 1.5σ level, while the *F*_o_ − *F*_c_ map (red) is shown at −3.0σ.

Whereas our analysis primarily concerned structures in which water molecules were missing, we also found cases where water molecules were listed in the deposited coordinate files but the corresponding electron-density maps did not support their presence. Thus, for example, the structure of the botulism neurotoxin light chain (PDB entry 7kyf; Amezcua *et al.*, 2021 ▸), determined at 2.33 Å resolution, contains 19 water molecules. All of them were modeled at full occupancy with ADPs of exactly 30.00 Å^2^, a *Coot* default, indicating that these parameters were not refined. However, these water molecules are all located in difference electron-density peaks lower than −3σ (Fig. 5 ▸), suggesting that their placement was not based on the available diffraction data. An analogous phenomenon is seen in PDB entry 7ky2 but not in PDB entry 7kyh, with no water molecules modeled in the latter case. All three structures are described in the same publication (Amezcua *et al.*, 2021 ▸). The depositors were notified by us directly about these problems but had taken no action to ameliorate them as of the time of completion of this manuscript.

**Figure 6:**
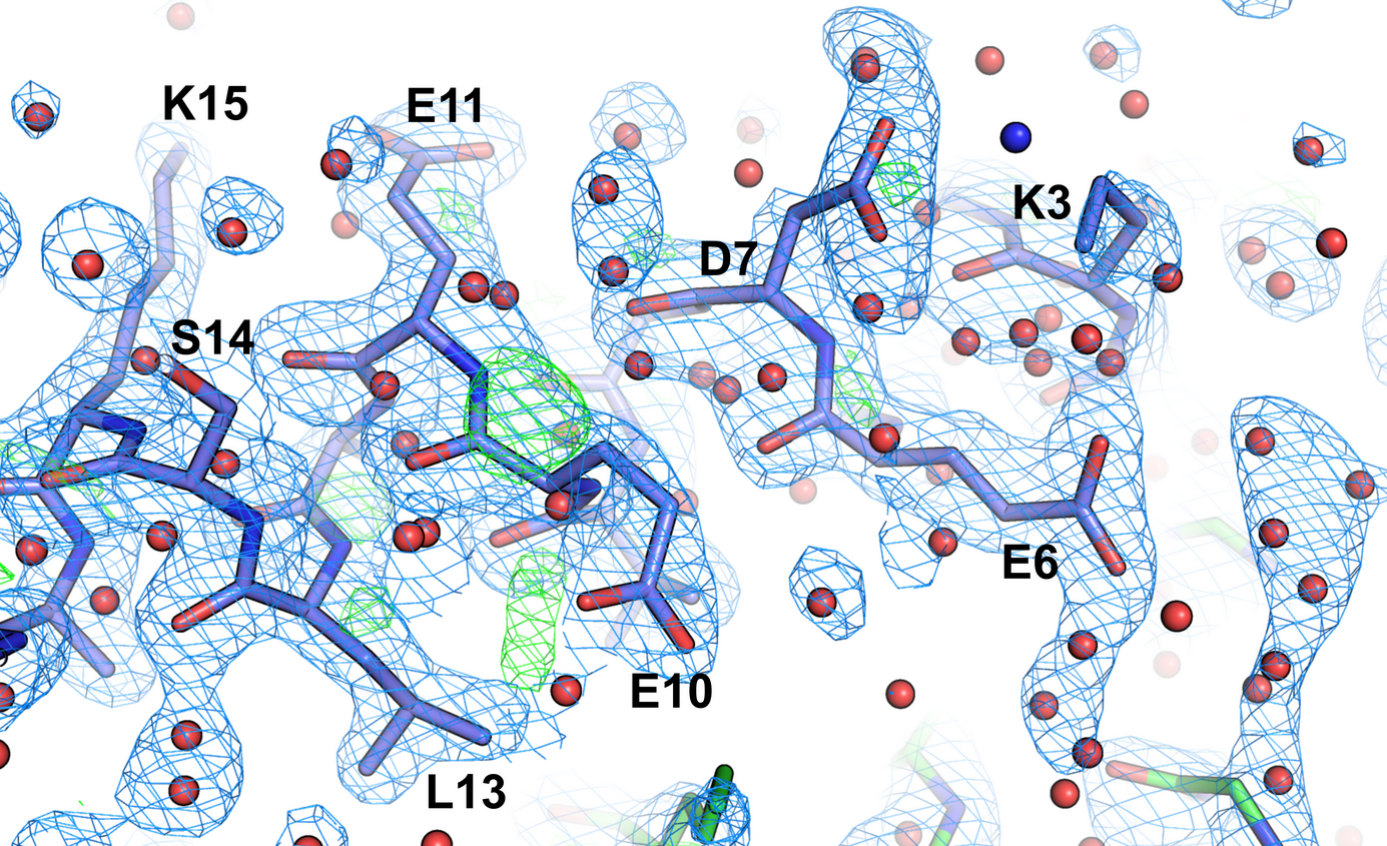
Structure of the GCN4 leucine-zipper core mutant N16A (PDB entry 3k7z, chain *B*) with a large excess of water molecules that are not located in significant positive peaks in the electron-density maps and are overlapping the protein coordinates. The 2*F*_o_ − *F*_c_ map (blue mesh) is shown at the 1.5σ level, whereas the *F*_o_ − *F*_c_ map is shown at 3.0σ (green). Water molecules are shown as red spheres; the blue sphere is the NZ atom of the incorrectly modeled Lys3 (suggesting incorrect geometric restraints).

A perplexing case involving a large excess of water molecules is presented by the 1.9 Å resolution structure of the GCN4 leucine-zipper core mutant N16A (PDB entry 3k7z; Holton & Alber, 2004 ▸). The asymmetric unit of the trigonal unit cell contains 93 residues accompanied by 621 water molecules. A vast majority of these water molecules are not located in any significant positive peaks in the 2*F*_o_ − *F*_c_ electron-density map and many are overlapping the coordinates of the protein (Fig. 6 ▸). What is truly puzzling is that this PDB entry is a replacement for an earlier deposition, PDB entry 1rb1, in which the coordinates and *B* factors (ADPs) of the protein model are identical to the redeposition, but the solvent model is not, although the number of water molecules stayed the same in both depositions. This structure resulted from an experimental procedure for automated structure determination and refinement. However, at the very least, the fact that the solvent model is not related to the experimental data should be made very clear to the users of this PDB entry.

### 3.5. Structures with unusual occupancies and ADPs of solvent molecules

The ratio of the number of reported solvent molecules to that of protein residues as a function of resolution is presented in Fig. 1 ▸(*b*). Any substantial deviation from the expected number of solvent molecules should be carefully inspected before the protein model is declared to be refined. The number of solvent molecules is easy to find in the validation report. However, there are 400 structures that report waters that have zero occupancy and 190 that report waters with occupancy lower than 0.1, which, from the experimental point of view, is equivalent to zero. It is essential to realize that the result of the diffraction experiment is the electron-density map. A macromolecular model represents an interpretation of the electron-density map. Consequently, atoms with zero occupancy are present only in the model, which may mislead some bio­medical scientists who examine the model but do not examine the electron-density maps. At the other end of the spectrum are waters with occupancies between 0.9 and 0.99; we identified over 3500 such structures in the PDB (Supplementary File S4). With the exception of structures determined at extremely high resolution, it is unlikely that such estimates of occupancy could be accurate, especially since this parameter is closely correlated with the *B* factors in the refinement procedure. The presence of more than 100 structures in which the occupancy of water molecules exceeds 1.0 is even more difficult to explain.

An interesting example of a structure in which the occupancies of many water molecules have been refined is provided by a recent 1.8 Å resolution structure of SARS-CoV-2 main protease bound to an inhibitor (PDB entry 6ynq). Currently, one can examine the sixth version of this deposition. The refined water occupancies are in the range of 0.58–1.0, with many in the range of 0.95–0.99. This is a clear example of overfitting, as the number of parameters used in the refinement was unnecessarily excessive. This example also shows the limitations of *PDB-REDO* in handling solvent structure. *PDB-REDO* removed 87 out of 300 water molecules in PDB entry 6ynq, but did not correct the occupancies of the remaining solvent molecules. Table 1 ▸ lists the first 20 water molecules from PDB entry 6ynq. Seven water molecules that were removed by the *PDB-REDO* system are shown in italics, but the occupancies of other water molecules remained the same.

## 4. Discussion

For the integrity and maintenance of their native three-dimensional structure, globular proteins must always be surrounded by a shell of water molecules, called the sphere of hydration (Virtanen *et al.*, 2010 ▸). The sphere of hydration may be divided into one or more layers. The first layer of hydration is always very tightly associated with the protein molecule and well ordered. Farther away, the layers of hydration fade into disordered bulk solvent. Occasionally, water molecules are also found occluded in the hydrophobic protein interior and/or at other critical points, such as active sites or metal-coordination spheres. Consequently, protein crystals grown from aqueous solutions will always contain water of hydration. The fact that without their intrinsic water of hydration protein crystals will cease to exist was noted at the inception of protein crystallography (Bernal & Crowfoot, 1934 ▸; Bernal *et al.*, 1938 ▸; Crowfoot & Riley, 1939 ▸). The volume fraction occupied by solvent varies from crystal to crystal, as elegantly worked out by Matthews (1968 ▸), but at least some well ordered water molecules are present in all protein crystals, including those of very small proteins or polypeptides. The typical hydrogen-bonding distances of the water molecules in the first hydration layer are ∼2.8 Å (Jiang & Brünger, 1994 ▸). The visualization of such water molecules in X-ray crystal structures (and also in cryoEM maps) will, therefore, depend on the resolution of the diffraction data. At resolutions lower than 3 Å, mapping water molecules will be difficult. However, if the map resolution is better than 2.75 Å, a variable number of water molecules will emerge in the maps, but some will always be discernible.

The analysis presented here clearly shows that the absence of water molecules in crystal structures determined at resolutions higher than ∼2 Å is an indication of problems with either the diffraction data, the refinement or the deposition process (or often a combination thereof). For structures at a resolution in the range 2–2.5 Å, the lack of water molecules may simply reflect the poor quality of the diffraction data, but it is a flag that raises questions about the overall quality of that particular deposition.

In this paper, we have exposed the curious fact that even protein crystal structures at ultimate resolution and/or determined explicitly to study the water networks have been deposited in the PDB without any water molecules. This surprising defect, certainly confounding any analyses based on such PDB entries, is not an isolated phenomenon. Even using very rigorous filters, we were able to find nearly 800 waterless protein crystal structures in the PDB that were determined at a resolution of 2.5 Å or higher. Such incomplete entries are very often marred with additional defects, such as inconsistency of the *R* factors reported by the depositors and the DCC, an incorrect *R*/*R*_free_ relationship, *etc.* In another category are depositions with long lists of water molecules with zero occupancy. An even more puzzling curiosity is a deposition where water O atoms have 0.0 occupancies but refined ADP factors, whereas the H atoms of the same water molecules are present with occupancies of 1.0 but ADPs of 0.0 Å^2^. Not much better, and indeed even more confusing, are depositions where water molecules are included with minuscule occupancy (≤0.1). Also illogical is the other end of the scale, where water molecules are assigned near-full occupancy (*e.g.* 0.99). Such water molecules should, of course, be modeled with occupancy 1.0.

The popular graphics program *PyMOL* (DeLano, 2002 ▸), when used in default mode (which is the most likely mode in the hands of biomedical researchers who are not structural biologists), displays all atoms in the same way, regardless of their occupancy parameter. That means that atoms with zero (or even negative) occupancy will be portrayed as fully legitimate atoms, leading to very precarious situations when PDB structures with such atoms are analyzed and interpreted using this software package.

The most crucial difference between the *PDB-REDO* server (Joosten *et al.*, 2011 ▸), created to re-refine all X-ray structures deposited in the PDB, and the PDB itself, is that *PDB-REDO* can modify structures. In contrast, the PDB can only send validation reports to depositors or use the CAVEAT option to caution users about problematic issues. Unfortunately, *PDB-REDO* was unable to correct the issue of missing water molecules, at least for the cases that we checked manually. It is even more troubling that in some cases remedying structures with *PDB-REDO* makes things even worse. Examples are provided by PDB entries 7t4u, 7t4v and 7t4w, where *PDB-REDO* elevated the occupancy of all water molecules to between 2.00 and 9.00. This highlights the need to address the problems with water modeling in PDB depositions even more strongly. We have already contacted the PDB and *PDB-REDO* directly to signal these problems. We also hope that the analysis and conclusions presented in this paper will help the PDB curators to make this database an even better resource.

The importance of water structure cannot be overestimated, as scientists retrieve structural information from the PDB for various purposes, including further structural studies, drug discovery, molecular modeling, drug design, tweaking catalytic performance and detailed understanding of protein function. Water molecules usually form hydrogen bonds with polar or charged groups of the macromolecule, stabilizing the secondary, tertiary and quaternary structures of proteins. Moreover, water molecules can mediate interactions between proteins and ligands, often forming hydrogen bridges that facilitate binding. Thus, the modeling and refinement of water molecules is as crucial as the refinement of proteins, protein–ligand interactions *etc.* One can argue that a water occupancy of 0.98 is as legitimate as 1.0; however, even diffraction data extending to a resolution of 1 Å or higher do not justify distinguishing between occupancies that differ by 2%. Thus, occupancy refinement should be performed only for high-resolution diffraction experiments, much higher than for PDB entry 6ynq, the example discussed above. Sometimes structure depositors overuse occupancy or multiple conformations to justify disagreement between model and electron density or between the interpretation of nondiffraction experiments and structural models derived from X-ray diffraction data.

Omitting important solvent molecules from the deposited model may lead to subsequent misinterpretations, as exemplified by a recent case. In PDB entry 6m0c very clear water molecules observed in the electron density were left unassigned and a particularly significant water molecule was not present in the model. The same structure was later determined at room temperature, and the authors of the new structure argued that the crucial catalytic water can only be observed at physiological temperature (Kneller *et al.*, 2020 ▸). A comparison of the electron density for both depositions shows that in this respect there is no difference between the structures determined at 100 K and at room temperature.

The example above shows that the goal of structural biology experiments (the interpretation of the electron-density maps and structure refinement) should be a model that is closest to reality and not necessarily the pursuit of the lowest *R* factors. The water structure is a significant part of the structural model and should be carefully refined. Solvent molecules with zero occupancy should be removed from the model, or the vicinity of such a molecule should be carefully re-examined and re-refined. The number of suspicious water molecules in Table 2 ▸ may be surprising. However, it has previously been shown (Raczynska *et al.*, 2018 ▸) that manipulation of the occupancy parameters or adding double conformations of side chains or ligands may sometimes be used to present wishful thinking as legitimate scientific results.

We would like to stress that we are not critical of the PDB or fail to recognize the immense value that this repository brings to the biomedical community. On the contrary, we have the utmost appreciation for the PDB and firmly believe that it is an exceptional resource that has transformed the fields of structural and molecular biology, as well as drug discovery. Since its establishment in 1971, the PDB has consistently evolved, integrating new technologies while upholding the highest standards of data accuracy and accessibility.

It is true that PDB depositions can sometimes be less than ideal or even contain significant errors, but these mistakes are typically the responsibility of the depositors. One may wish that the PDB would develop even better validation tools and a quicker error-handling process. The PDB remains an unparalleled resource compared with other databases. Given the complexity of the work and the human factor involved, the occasional errors that we encounter are unavoidable. We actively collaborate with the PDB, reporting many of these errors to help to maintain the high standard that the entire scientific community relies upon.

**Figure 7:**
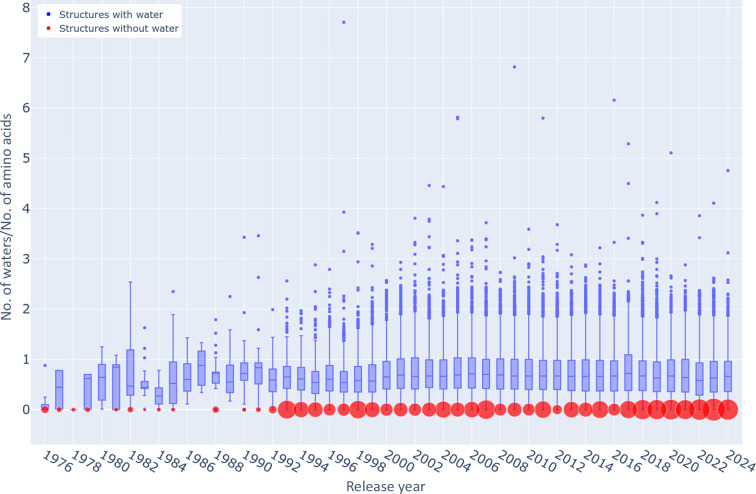
Ratio of the number of water molecules to the number of amino-acid residues in structures deposited each year in the PDB at resolutions of 2.5 Å or higher. The limits of the blue boxes are at the first and third quartiles and the line within each box represents the median. The lower and upper whiskers mark the distance of 1.5 interquartile ranges from those quartiles. The individual dots show outliers. An interactive version of this figure (available at https://bioreproducibility.org/figures/water_paper/fig7) enables the identification of each outlier. The diameters of the red circles are proportional to the number of waterless structures deposited each year.

The importance of the PDB, not only as a source of structural information about individual proteins, but also as a source of training sets for computational methods that are exemplified by the various versions of *AlphaFold* (Jumper *et al.*, 2021 ▸) and other similar software, cannot be overemphasized. The download/access frequency of individual PDB depositions can vary widely depending on several factors, including the significance and novelty of the structure, its biological relevance and the interest of the research community in that particular protein or complex. However, maintenance of the highest overall quality of the database as a whole (with an understanding that individual structures should be accurate to the level allowed by the experimental data) is of paramount importance. The absence of water molecules or their unusual occupancies in high-resolution structures should be treated as a very red validation flag and prominently marked in order to prevent the use of such structures in data mining and global analyses. As pointedly illustrated in Fig. 7 ▸, the problem of depositions in the PDB with an unreasonable number of water molecules is not ephemeral or new but has persisted for many years. The number of waterless structures deposited each year has remained quite constant despite the leveling or even a decrease in the number of crystal structures released each year, especially during the last decade (Table 3 ▸). The only way to ameliorate this situation is through better vigilance on the part of the PDB, with special attention paid in the validation procedures and reports to the solvent component in medium-to-high-resolution crystal structures (and ultimately of cryoEM structures as well). To improve current practices, the PDB validation report should be expanded to provide clearer alerts regarding quality issues. Specifically, information about unusual water counts and occupancies (whether too high or too low) should be included as an additional metric on the structure-quality slider. We have already informed the PDB about this concern.

Addressing the issue of existing depositions that lack adequate water representation presents a different challenge. While the PDB may implement better validation procedures that would alert depositors about serious problems with solvent structure, the PDB itself is not in the position to enforce the required corrections, let alone to implement them. We do hope that the *PDB-REDO* server can be enhanced not only to eliminate erroneous water molecules, but also to recognize waterless structures and actively build a correct solvent model. Ultimately, however, the onus of proper modeling of water structure really falls on depositors, especially supervisors and leading authors. We should all understand that PDB deposition is not just a nuisance required by journals, but that it is a vital part of the process of discovery. By depositing the best possible models, and this includes the solvent structure as well, we in a sense stamp a seal of quality, which will be especially important when the database is used for automatic large-scale data mining. Finally, proper mentoring and supervision in this respect is an opportunity to instill into the minds of the next generation the notion that we all have responsibilities as crystallographers and scientists. This, by the way, also applies to the other issues raised in this article, such as data processing, refinement protocols, validation and the deposition process itself. We hope that the points that we have raised might lead to a discussion in the community that will ultimately result in improved quality of the deposited structural data. 

## Supplementary Material

Supplementary File S1. The final working data set. DOI: 10.1107/S2052252524009928/be5300sup1.xlsx

Supplementary File S2. Statistics from electron-density maps. DOI: 10.1107/S2052252524009928/be5300sup2.xlsx

Supplementary Figure S1. Results of agglomerative clustering. DOI: 10.1107/S2052252524009928/be5300sup3.png

Supplementary File S3. Waterless structures with the highest outlier factors. DOI: 10.1107/S2052252524009928/be5300sup4.xlsx

Supplementary File S4. Structures with waters with occupancies of between 0.9 and 0.99. DOI: 10.1107/S2052252524009928/be5300sup5.xlsx

## Figures and Tables

**Table 1:** The first 20 water molecules from PDB entry 6ynq The column containing occupancies is highlighted in bold. Water molecules with fractionally refined occupancy are shown in red. The seven water molecules that were removed by *PDB-REDO* are shown in italics.

Record name	Serial No.	Atom name	Residue name	Chain	Residue ID	*x*	*y*	*z*	Occupancy	Temperature factor (Å^2^)	Element
HETATM	5046	O	HOH	*A*	501	21.069	−3.189	32.245	**0.85**	48.76	O
*HETATM*	*5047*	*O*	*HOH*	*A*	*502*	*21.656*	*−2.374*	*25.903*	***0.58***	*38.42*	*O*
*HETATM*	*5048*	*O*	*HOH*	*A*	*503*	*12.136*	*30.803*	*−8.812*	***0.78***	*36.68*	*O*
HETATM	5049	O	HOH	*A*	504	16.659	−5.561	33.251	**1.00**	47.52	O
*HETATM*	*5050*	*O*	*HOH*	*A*	*505*	*16.365*	*31.436*	*0.767*	***0.80***	*47.21*	*O*
HETATM	5051	O	HOH	*A*	506	15.871	−13.121	31.723	**1.00**	40.38	O
*HETATM*	*5052*	*O*	*HOH*	*A*	*507*	*13.213*	*23.824*	*−18.522*	***1.00***	*55.50*	*O*
HETATM	5053	O	HOH	*A*	508	6.749	−1.420	−4.404	**0.71**	33.78	O
*HETATM*	*5054*	*O*	*HOH*	*A*	*509*	*3.663*	*3.997*	*−18.333*	***0.93***	*47.53*	*O*
HETATM	5055	O	HOH	*A*	510	4.326	−6.611	−17.259	**1.00**	49.70	O
HETATM	5056	O	HOH	*A*	511	0.035	−1.289	12.377	**0.78**	28.89	O
HETATM	5057	O	HOH	*A*	512	19.251	−23.552	9.203	**0.88**	44.06	O
HETATM	5058	O	HOH	*A*	513	6.327	10.284	−0.879	**1.00**	25.60	O
*HETATM*	*5059*	*O*	*HOH*	*A*	*514*	*1.973*	*14.147*	*0.488*	***0.87***	*36.33*	*O*
HETATM	5060	O	HOH	*A*	515	10.913	−16.850	29.957	**0.89**	41.88	O
HETATM	5061	O	HOH	*A*	516	3.010	5.399	20.334	**1.00**	29.04	O
HETATM	5062	O	HOH	*A*	517	14.798	−25.987	26.809	**1.00**	30.70	O
HETATM	5063	O	HOH	*A*	518	15.797	−21.945	31.403	**0.99**	29.00	O
*HETATM*	*5064*	*O*	*HOH*	*A*	*519*	*24.445*	*−5.903*	*8.988*	***0.86***	*38.58*	*O*
HETATM	5065	O	HOH	*A*	520	3.655	22.359	−0.802	**0.92**	42.03	O

**Table 2:** The number of crystal structures containing water molecules with highly unusual occupancies

Water occupancy	Structures (PDB)	Structures (*PDB-REDO*)	Water molecules (PDB)	Water molecules (*PDB-REDO*)
0.0	400	150	3581	406
0.01–0.1	190	81	1342	118
>1.0	109	27	3819	901

**Table 3:** The numbers of protein X-ray crystal structures (resolution higher than or equal to 2.5 Å) released each year by the PDB and of those without water molecules

Release year	No. released	No. waterless	Percentage waterless
1995†	2477	127	5.13
1996	664	16	2.41
1997	922	17	1.84
1998	1346	33	2.45
1999	1498	25	1.67
2000	1763	17	0.96
2001	1876	21	1.12
2002	1975	17	0.86
2003	2831	22	0.78
2004	3506	29	0.83
2005	3426	22	0.64
2006	4395	27	0.61
2007	4846	37	0.76
2008	4840	16	0.33
2009	5236	22	0.42
2010	5519	16	0.29
2011	5640	30	0.53
2012	6202	10	0.16
2013	6545	27	0.41
2014	6564	22	0.34
2015	6326	31	0.49
2016	7136	16	0.22
2017	6765	30	0.44
2018	7106	24	0.34
2019	7124	40	0.56
2020	7615	40	0.53
2021	6688	38	0.57
2022	6995	45	0.64
2023	6632	50	0.75
2024‡	6376	43	0.67

† The data up to 1995 were accumulated and are shown in one row.

‡ The data for 2024 are extrapolated, based on the data as of 6 October 2024, to reflect the full year.
