# Supplementary figures and images for: Waterless structures in the Protein Data Bank
Source: IUCrJ. 2024 Oct 28;11(Pt 6):966–76. doi: 10.1107/S2052252524009928 (PMC11533996; doi:10.1107/S2052252524009928)

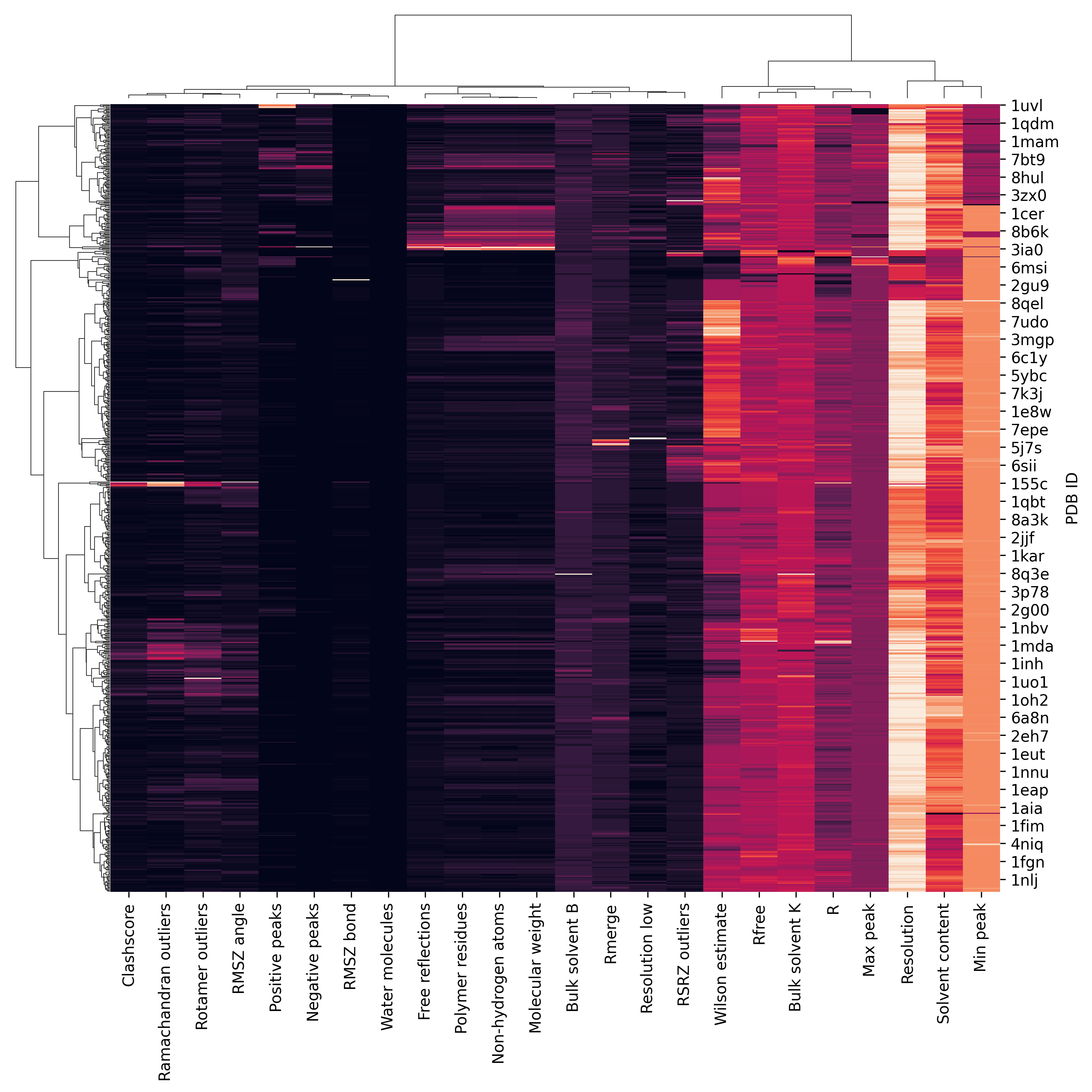

Supplement: Supplementary file 3 [file m-11-00966-sup3.png]
